# Oestrogen-induced angiogenesis promotes adenomyosis by activating the Slug-VEGF axis in endometrial epithelial cells

**DOI:** 10.1111/jcmm.12300

**Published:** 2014-04-24

**Authors:** Tze-Sing Huang, Yi-Jen Chen, Teh-Ying Chou, Chih-Yao Chen, Hsin-Yang Li, Ben-Shian Huang, Hsiao-Wen Tsai, Hsin-Yi Lan, Cheng-Hsuan Chang, Nae-Fang Twu, Ming-Shyen Yen, Peng-Hui Wang, Kuan-Chong Chao, Chun-Chung Lee, Muh-Hwa Yang

**Affiliations:** a National Institute of Cancer Research, National Health Research InstitutesMiaoli, Taiwan; b Department of Obstetrics and Gynecology, Taipei Veterans General HospitalTaipei, Taiwan; c Institute of Clinical Medicine, National Yang-Ming UniversityTaipei, Taiwan; d Department of Pathology, Taipei Veterans General HospitalTaipei, Taiwan; e Department of Obstetrics and Gynecology, National Yang-Ming University HospitalIlan, Taiwan; f Division of Hematology-Oncology, Department of Medicine, Taipei Veterans General HospitalTaipei, Taiwan; g Immunology Research Center, National Yang-Ming UniversityTaipei, Taiwan

**Keywords:** adenomyosis, angiogenesis, epithelial–mesenchymal transition, oestrogen, Slug

## Abstract

Adenomyosis is an oestrogen-dependent disease characterized by the invasion of endometrial epithelial cells into the myometrium of uterus, and angiogenesis is thought to be required for the implantation of endometrial glandular tissues during the adenomyotic pathogenesis. In this study, we demonstrate that compared with eutopic endometria, adenomyotic lesions exhibited increased vascularity as detected by sonography. Microscopically, the lesions also exhibited an oestrogen-associated elevation of microvascular density and VEGF expression in endometrial epithelial cells. We previously reported that oestrogen-induced Slug expression was critical for endometrial epithelial–mesenchymal transition and development of adenomyosis. Our present studies demonstrated that estradiol (E2) elicited a Slug-VEGF axis in endometrial epithelial cells, and also induced pro-angiogenic activity in vascular endothelial cells. The antagonizing agents against E2 or VEGF suppressed endothelial cells migration and tubal formation. Animal experiments furthermore confirmed that blockage of E2 or VEGF was efficient to attenuate the implantation of adenomyotic lesions. These results highlight the importance of oestrogen-induced angiogenesis in adenomyosis development and provide a potential strategy for treating adenomyosis through intercepting the E2-Slug-VEGF pathway.

## Introduction

Adenomyosis is defined as the presence of endometrial glandular tissue in the myometrium and smooth muscle hyperplasia, which remains an important cause of menorrhagia, dysmenorrhoea and preterm labour in women of reproductive age [1,2]. Regarding the pathogenic mechanism of adenomyosis, strong evidence suggests that the migratory and invasive properties of endometrial epithelial cells can be induced by oestrogen [3–5]. In addition, neovascularization is also considered to be another major pathological feature of adenomyosis/endometriosis [6–8]. Angiogenesis is required for the invasion of ectopic endometrial implants into pelvic sites and their subsequent proliferation [6,7,9]. VEGF, a major angiogenic factor, is an important protein for angiogenesis and thus the development of adenomyosis [8]. However, how these endometrial cells acquire the invasive and pro-angiogenic phenotypes simultaneously and what key regulator(s) underlie the pathogenesis under oestrogen stimulation remain unclear.

Accumulated evidence supports the role of oestrogen in the angiogenesis of normal uterine tissues [9]. In a normal uterus, oestrogen is required for the development of endometrial vasculature during the menstrual cycle. A marked reduction in VEGF levels was shown in the endometrial glandular epithelial and stromal cells after oophorectomy of baboons and rhesus monkeys [10,11], and administration of estradiol (E2) restored VEGF expression and microvascular permeability, which are the early events of angiogenesis [9]. VEGF is inducible by oestrogen in endometrial epithelial cells [12], and can also be intensively expressed in the endometrial glandular tissues [13,14]. Moreover, vascular endothelial cells express oestrogen receptor (ER) [15], and are responsive to oestrogen stimulation for cell proliferation and development into new blood vessels [9,16]. However, the pathogenic role of oestrogen-stimulated angiogenesis in the development of adenomyosis remains unclear.

Epithelial–mesenchymal transition (EMT) is a process by which epithelial cells lose their polarity and acquire the mesenchymal phenotype, and this mesenchymal reprogramming is critical for the acquired invasiveness of epithelial cells [17]. In addition to the acquisition of invasiveness, the role of EMT in angiogenesis has been attracting more attention. The EMT regulators, Twist and Slug are critical in Myc-induced vascular development in Xenopus [18]. In breast cancer, Twist facilitates cancer cell, VEGF expression and promotes tumour angiogenesis [19]. Snail has also been shown to induce angiogenesis in Madin-Darby canine kidney epithelial cells [20]. Although EMT has been well recognized as a key mechanism in embryogenesis and development of certain malignancies [21], whether EMT also plays a crucial role in the angiogenesis of adenomyosis is unknown.

We previously reported that oestrogen induces EMT in endometrial epithelial cells by up-regulating Slug expression and that this process is critical for the development of adenomyosis [5,22]. In this study, we furthermore demonstrate the role of oestrogen-induced angiogenesis in the development of adenomyosis through the activation of Slug-VEGF axis.

## Materials and methods

### Study population, sample collection and the pathological diagnosis of adenomyosis

This protocol was approved by the Institutional Review Board of Taipei Veterans General Hospital (VGHIRB No. 2012-02-015A). Adenomyosis lesions and the corresponding eutopic endometria samples were collected during hysterectomy from 106 women with adenomyosis without a history of oral contraceptive use within 3 months. To prevent the impact of tissue quality on staining results, six cases were removed from the study because of inadequate tissue quality, as determined by the gynaecological pathologist. Finally, 100 women with adenomyosis were enrolled in this study. Endometrial tissues were obtained from 30 women with normal menstrual cycles undergoing hysterectomies for benign gynaecological conditions (uterine prolapse, ovulatory dysfunctional uterine bleeding, benign ovarian tumour or cervical intraepithelial neoplasia) other than endometrial disease to serve as controls. We also confirm that there is no endometriosis case in the study and control group. The women's menstrual cycle phases were established based on their menstrual history and confirmed by endometrial histology using the criteria previously described [23]. The early proliferative phase was defined as the 4th–7th day of the menstrual cycle, the mid-proliferative phase was defined as days 8–10 and the secretory phase was defined as previously described [24]. In this study, all of the patients were in the secretory or the early and mid-proliferative phase [24].

### Blood flow in the myometrium of women with or without adenomyosis by three-dimensional power Doppler sonography

All data from adenomyosis patients were acquired using a Voluson 730 (GE Medical Systems, Zipf, Austria) ultrasound machine equipped with a 2.8–10 MHz transabdominal and a 5 MHz transvaginal transducer before hysterectomy. Adenomyosis was diagnosed based on the pathological report. Finally, 100 women with adenomyosis without associated pathology were enrolled in this study. Thirty women (uterine prolapse, dysfunctional uterine bleeding, benign ovarian tumour or cervical intraepithelial neoplasia) without myoma, adenomyosis, endometriosis or endometrial lesions served as controls. For 3D ultrasound, we scanned transabdominally. Once a satisfactory greyscale image (longitudinal view) of the uterus had been obtained, the uterus was centralized onscreen, and a 3D power Doppler data set of the uterus was acquired, ensuring that a complete volume of the myometrial and endometrial area had been captured. The vascularization index (VI) and vascularization flow index (VFI) were calculated using colour power Doppler angio and the VOCALTM (virtual organ computer-aided analysis) software for histogram analysis once the total volume of the uterus was stored, excluding the peripheral supplying vessels [25,26]. The VI, measuring the ratio of the number of colour voxels to the number of all voxels, is thought to represent the density of blood vessels (vascularity) and was expressed as a percentage (%) of the uterine volume. The VFI is a combination of vascularization and blood flow information; thus, it represents overall perfusion [27,28]. The VFI refers to the weighted colour voxel/total voxel ratio, combining the information of vessel presence (vascularity) and amount of transported blood cells (blood flow). The VFI = VI × FI (VI mutiplied by FI) [29]. All Doppler measure-ments were performed by one examiner to prevent the inter-operator variations (Dr. Chih-Yao Chen of Taipei Veterans General Hospital).

### Immunohistochemistry (IHC)

The IHC for formalin fixed, paraffin-embedded samples was performed as described [30]. The samples were cut in 6-μm sections on poly-l-lysine-coated slides. Following deparaffinization with xylene (10 min. twice), sections were rehydrated through decreasing concentrations of ethanol (100% ×2 times, 95%, 85% and 70%) and washed for 5 min. For antigen retrieval, the specimens were heated in 10 mM citrate buffer (pH 6.0) using a microwave for 12 min. For frozen specimens from dissected tumours in nude mice, 6-μm thick sections of tumour were cut and fixed in acetone, air-dried, and subsequently bathed in Tris-buffered saline solution (pH 7.6). The endogenous peroxidase activity was blocked with 3% hydrogen peroxide. Antibody characteristics are listed in Table S1 [5,31].

### Quantification of IHC results

The IHC slides were independently examined by two observers. We evaluated the VEGF and Slug expression in the basal layer of normal and eutopic endometrium and ectopic endometria. We utilized immunohistochemical scores (IHS) based on the German ImmunoReactive score [32]. The IHS was calculated by combining an estimate of the percentage of immunoreactive cells (quantitative score) with an estimate of the staining intensity (staining intensity score). No staining was scored as 0; staining of 1–10% as 1; 11–50% as 2; 51–80% as 3 and 81–100% as 4. The staining intensity was rated on a scale of 0 to 3, with 0 representing no staining, 1 weak staining, 2 moderate staining and 3 strong staining. The raw data were converted to IHS by multiplying the quantity score and the intensity score. A moderate to strong level (IHS >4) of VEGF and Slug expression was considered to be positive, and weak or absent expression (IHS 0–4) was considered to be negative. Microvascular density (MVD) was determined in the basal layer of normal and eutopic endometrium and ectopic endometria by counting all of the vessels at 400× magnification within an examination area of 0.22 mm^2^. Each stained lumen was regarded as a single countable microvessel. If only a single CD31-positive cell was visible with no lumen, this cell was also interpreted as a microvessel. Values were expressed as the number of vessels/mm^2^ [8].

### Cell culture and drug treatment

The ER-positive Ishikawa cells [Ishikawa; Human Asian endometrial adenocarcinoma, European Collection of Cell Cultures (ECACC) N. 99040201] and ER-negative Ishikawa cells (Ishikawa02 ER-; Human endometrial adenocarcinoma, ECACC No. 98032302) were cultivated in DMEM containing 10% foetal bovine serum (FBS). To prevent the confounding effect of endogenous steroids, cells were placed in phenol red-free DMEM containing 10% FBS for 48 hrs before drug treatment to remove endogenous steroids. Cells were then incubated in fresh medium (negative control), E2 (10^−6^ M; Sigma-Aldrich, St. Louis, MO, USA), DMSO (solvent for E2 and raloxifene), raloxifene (5 × 10^−7^ M; a selective ER modulator; Sigma-Aldrich) or E2 plus raloxifene for 24 hrs.

### Protein extraction and western blot analysis

Protein extractions and Western blot analyses were performed as previously described [31]. Antibody characteristics are listed in Table S1.

### Detection of VEGF expression by ELISA

To detect the VEGF secretion of ER-positive Ishikawa cells and ER-negative Ishikawa02 cells, the VEGF levels in the culture media were analysed by a sandwich ELISA (Quantikine; R&D Systems, Minneapolis, MN, USA) using monoclonal and polyclonal antibodies capable of detecting VEGF165/121. The results shown are representative of three independent experiments.

### Vascular endothelial cell capillary tube and network formation assay

The primary human umbilical vein endothelial cells (HUVECs) were cultivated as previously described [33]. HUVECs between passages 2 and 4 were used for the experiments. *In vitro* endothelial cell tube and network formation assays were used to evaluate the pro-angiogenic activity of E2 in the absence or presence of raloxifene or bevacizumab (Bevacizumab, anti-VEGF antibody). Matrigel (BD Bioscience, San Jose, CA, USA) was thawed and diluted with an equal volume of M199 medium. The mixtures were transferred to a 24-well plate and allowed to solidify by incubation at 37°C for 30 min. HUVECs (8 × 10^4^) in 200 μl medium containing E2 with or without raloxifene or bevacizumab were overlaid on the matrigel and incubated at 37°C for 3–4 hrs. The number of endothelial cell tubes and networks were recorded by microscopic photography and were quantified by measuring the total lengths of capillary tubes [33].

### Endothelial cell migration assay

Human umbilical vein endothelial cells (4 × 10^5^) were seeded onto a well of a 6-well plate for overnight. After cells had adhered completely, we scraped the cells with three horizontal lines and three vertical lines to make nine crosses in each well by a yellow tip. The width of each line scraped by yellow tip was ∼250–280 μm. After the lines were scraped, the cells were washed with PBS and were incubated for 16 hrs with the medium containing E2 in the absence or presence of 1, 5 or 10 nM of raloxifene or 0.5 mg/ml bevacizumab (bevacizumab, an anti-VEGF antibody). At the starting time and after 16 hrs, each cross was photographed at 100× magnification and the area of cross was measured using the software Image-Pro Plus version 5.0.2 (MediaCybernetics Inc., Silver Spring, MD, USA). The migration ability was calculated by measuring the area of the cross and using the formula: (1 − Area_16h_/Area_0h_) × 100% [34]. In addition, cell migration was also monitored for 16 hrs by time-lapse photography [35]. Twenty cells in each treatment group were randomly selected, and their migration tracks were analysed by image-Pro Plus software [35]. The accumulated and oriented distances of the migration tracks were further quantified and expressed as mean ± SD. The results shown are representative of three independent experiments.

### Plasmids construction and short hairpin RNA experiment

The plasmid pcDNA3-SLUG was generated by the insertion of an 807-bp fragment of the full-length human SLUG cDNA from the plasmid pCMVSPORT6-Snail2 (Genomic Center, National Yang-Ming University) into the BamHI/EcoRI sites of the pcDNA3.1 vector. For the short hairpin RNA (shRNA) experiment, the plasmid pSUPER-sh-Slug was generated by inserting an oligonucleotide containing shRNA target sequences specific to Slug into the pSUPER.puro vector. A scrambled sequence with no significant homology to any mammalian gene sequence was cloned into the pSUPER.puro vector (pSUPER-sh-scr) as a control for the siRNA experiments, as previously described [36]. The sequences of the oligonucleotides used to generate the shRNA constructs are listed in Table S2.

### Xenotransplantation of human adenomyosis lesions

Adenomyosis lesions and the corresponding eutopic endometria in the proliferative phase were obtained during hysterectomy from three pre-menopausal women with adenomyosis undergoing surgery at Taipei Veterans General Hospital. Fresh endometrial and adenomyosis tissue samples were fragmented into 1–2 mm diameter sections under sterile conditions. The fragments were cultured in DMEM + Ham's F12 (1:1) + 10% FBS supplemented with E2 (10^−9^ M) for 4 hrs prior to transplantation into non-obese diabetic severe combined immunodeficient (NOD-SCID) mice [37].

A total of 94 8-week-old NOD-SCID female mice were used. The guidelines for animal care were approved by the Committee on Animal Study of the Taipei Veterans General Hospital. The mice were bilaterally ovariectomized and left untreated for 14 days. Ten fragments of human adenomyosis lesions were implanted into the peritoneal cavity [5,38]. Drugs were delivered by subcutaneous injection. Sixty-four mice transplanted with adenomyosis fragments were injected with vehicle only (*n* = 10), E2 (0.2 μg/day; *n* = 34), raloxifene (0.1 μg/day; *n* = 10) or were co-treated with E2 and raloxifene (*n* = 10) [5]. Thirty mice transplanted with adenomyosis fragments were injected with vehicle only (*n* = 15), bevacizumab (5 mg/kg/day, three times per week; *n* = 15) [39]. Laparotomic examination was performed 21 days after implantation, and the mice were killed. The number of visible blood vessel branch points was statistically recorded for each mouse [40].

### Statistical analysis

Pearson chi-square or Fisher's exact tests were used for comparison of dichotomous variables. The independent Student's *t*-test or an anova was used to compare the continuous variables between groups. The level of statistical significance was set at 0.05 for all tests.

## Results

### Increased angiogenesis in adenomyotic tissues of the uterus

**Fig. 1:**
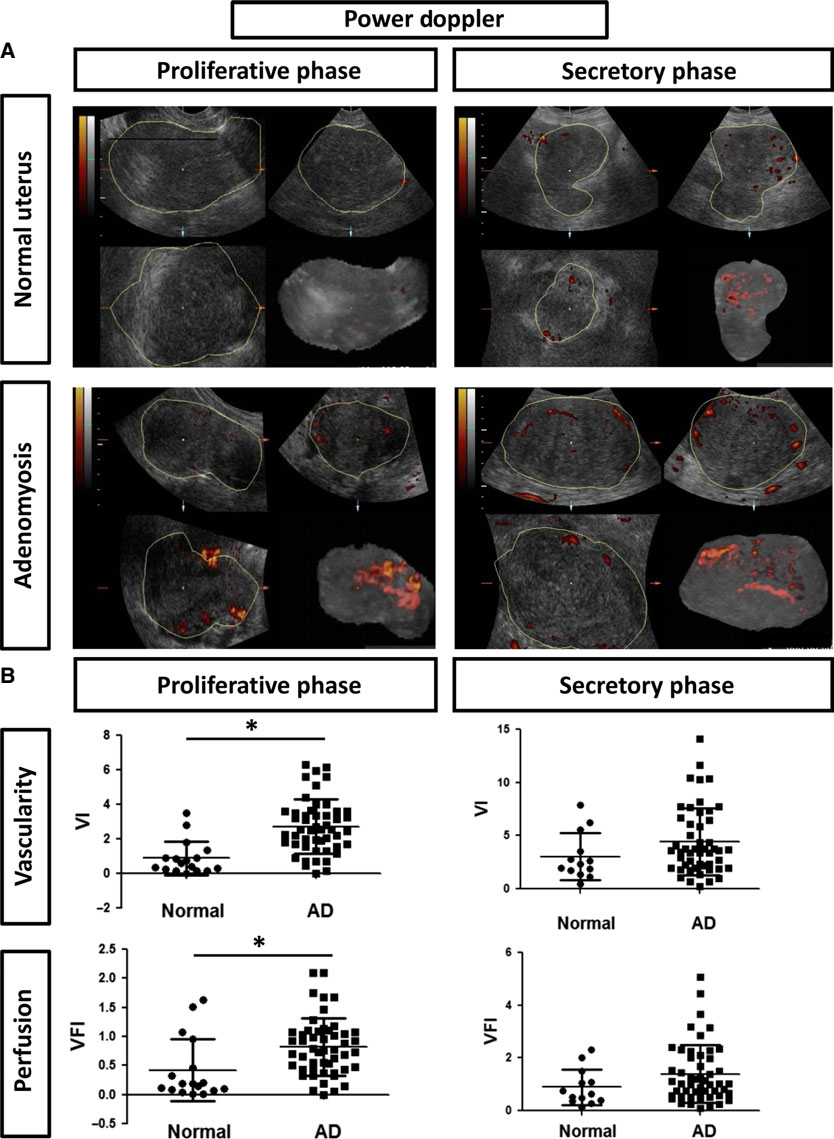
Analysis of the vascularity of the myometrium by Doppler sonography in women with or without adenomyosis during different menstrual phases. (**A**) The representative photos of the Doppler sonography in the proliferative *versus* secretory phase of the patients with adenomyosis or a normal control. The colourful Doppler signals for indicating blood flows are shown. (**B**) The contour histogram demonstrating the vascularity index (VI) and vascularity flow index (VFI) of the total myometrium in adenomyosis patients *versus* normal control. AD, adenomyosis. The asterisk (*) indicated statistical significance (*P* < 0.05).

To investigate the vascularity of adenomyosis tissue and the normal uterus, we analysed the results of three-dimensional power Doppler sonography in 100 patients with adenomyosis and 30 normal control cases before hysterectomy was performed. The characteristics of the adenomyosis patients are shown in Table S3, and the representative images of sonography are illustrated in Figure 1A. In adenomyosis patients, the uterus volume estimated by sonography was significant larger than that of a normal uterus (Table S3). The vascularity was estimated using two indexes, including the VI and VFI, which are thought to be related to the number of vessels within a given tissue volume. Compared with the control group, VI and VFI were significantly increased in adenomyosis patients in the proliferative phases (VI: control *versus* adenomyosis, 0.87 ± 1.01 *versus* 2.73 ± 1.56, *P* < 0.001; VFI: 0.44 ± 0.56 *versus* 0.82 ± 0.49, *P* = 0.013; Fig. 1B). Compared with the control group, VI and VFI in adenomyosis patients in the secretory phase approached, but did not reach statistical significance. (VI: control *versus* adenomyosis, 2.79 ± 2.18 *versus* 4.41 ± 3.15, *P* = 0.067; VFI: 0.81 ± 0.67 *versus* 1.40 ± 1.10, *P* = 0.056; Fig. 1B). This result indicates increased vascularity in the uterine lesion of adenomyosis patients.

**Fig. 2:**
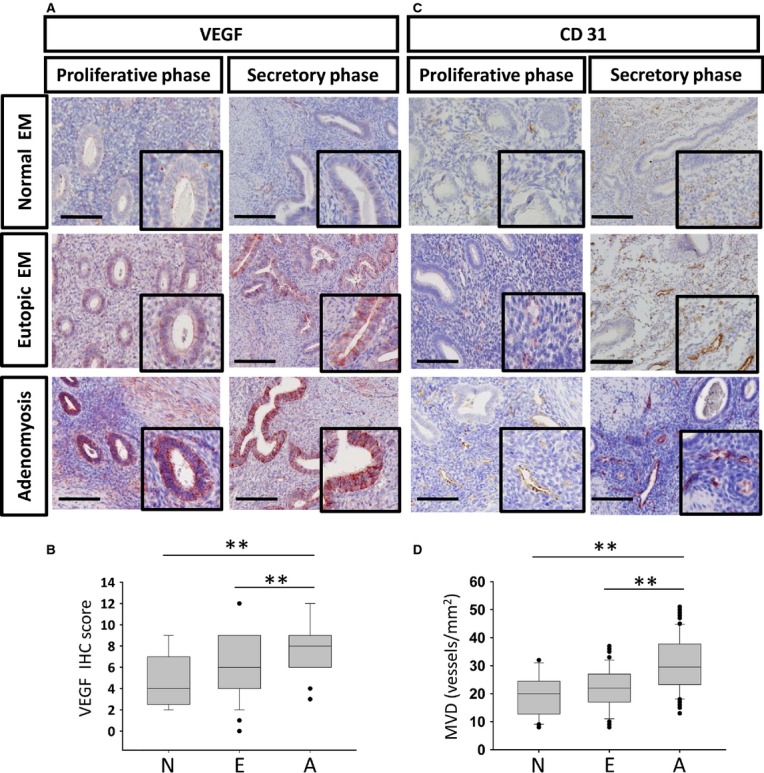
Analysis of the VEGF expression and microvascular density (MVD) in eutopic endometria and adenomyotic lesions of adenomyosis patients *versus* normal controls during different menstrual phases. (**A**) Immunohistochemistry for VEGF in normal endometria, eutopic endometria and adenomyosis during different phases of menstruation; scale bar = 800 μm. Original magnification: ×200 (upper); ×400 (lower). (**B**) The boxplots demonstrate the distribution of VEGF IHC scores in normal endometria (N), eutopic endometria (E) and adenomyosis (A). The asterisks (**) indicate statistical significance (*P* < 0.001). (**C**) Immunohistochemistry for CD31 in normal endometria, eutopic endometria and adenomyosis during different phases of menstruation; scale bar = 800 μm. Original magnification: ×200 (upper); ×400 (lower). (**D**) The boxplots demonstrate the distribution of MVD in normal endometria (N), eutopic endometria (E) and adenomyosis (A). The asterisks (**) indicate statistical significance (*P* < 0.001).

To confirm increased angiogenesis in adenomyotic tissues, we used IHC to examine the expression of VEGF and to determine the MVD by staining for an endothelial cell marker, CD31, in the samples from the previous patient population, which included 100 adenomyosis patients as well as 30 control cases who did not have adenomyosis but exhibited benign gynaecological diseases and were treated with a total hysterectomy. The patient characteristics of the adenomyosis patients are shown in Table S3. The results demonstrated that VEGF expression was present in eutopic endometria of adenomyosis patients compared with the endometria of normal control group, and a further increase in VEGF expression was noted in the endometrial epithelial cells of adenomyotic lesions (Fig. 2A and B). A significantly increased MVD was observed in adenomyotic lesions compared with the corresponding eutopic endometria and normal endometria, and normal endometria exhibited the lowest MVD (Fig. 2C and D). This result further confirms the development of angiogenesis in adenomyosis lesions.

### Increased vascularity in the secretory-phase uterus

Because the level of E2 is higher in the secretory phase compared with the early proliferative phase and E2 induces EMT in endometrial epithelial cells [5], we speculated the VEGF expression profile and MVD would be different among different menstrual phases. The distributions of the menstrual phases of the 100 patients and 30 control cases are summarized in Table S3. First, we confirmed the difference in E2 levels between the early proliferative and secretory phases of menstruation in normal and adenomyosis patients. The results demonstrated that in both normal control and adenomyosis patients, the serum E2 levels were relatively lower in patients who were in the early proliferative phase compared with those in secretory phase (Table S4). Next, we analysed the difference in vascularity between different menstrual phases by sonography. The result demonstrated that increased VI and VFI were observed in the secretory phase of the normal control group and adenomyosis patients compared with the proliferative phase (Fig. S1). Next, we investigated the expression of VEGF and MVD in samples during different phases of menstruation. Increased VEGF IHC scores were noted in the secretory phase compared with the early proliferative phase, and this effect was most prominent in eutopic endometria and the adenomyotic lesions of adenomyosis patients. Increased MVD was observed in the secretory phase of normal endometria, eutopic endometria and adenomyotic lesions compared with their early proliferative phase counterparts (Fig. S2).

Furthermore, we analysed the correlation between serum E2 levels and angiogenesis marker changes. A significant correlation was observed between serum E2 levels and VEGF positivity in normal and eutopic endometria and adenomyotic lesions (Fig. S3A–C). There was also a significant correlation between E2 levels and MVD in normal endometria, eutopic endometria and adenomyosis (Fig. S3D–F). Collectively, these results suggest the importance of E2 levels in promoting angiogenesis in uterine endometria.

### Oestrogen induces VEGF expression in both ER-harbouring endometrial cells and endothelial cells

**Fig. 3:**
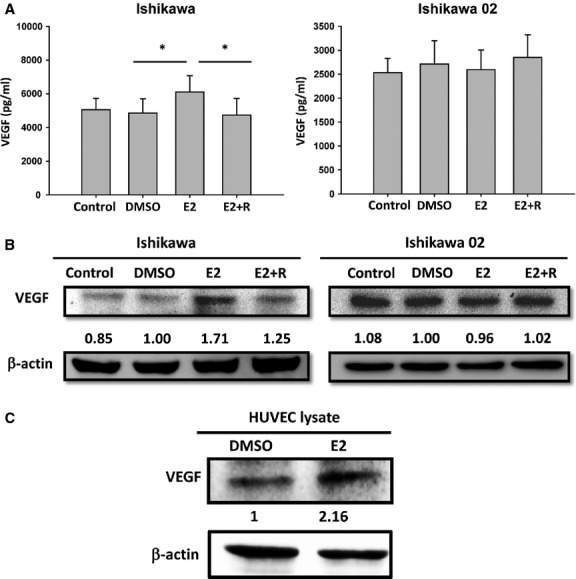
E2 induces VEGF expression/secretion in ER-positive Ishikawa and HUVEC cells but not in ER-negative Ishikawa02 cells. (**A**) ELISA for detecting the VEGF in the supernatants of ER-positive Ishikawa cells and ER-negative Ishikawa02 cells after treatment with E2 and E2 plus raloxifene (R) for 24 hrs. The histograms represent the mean ± SD of three independent experiments. The asterisk (*) indicates statistical significance (*P* < 0.05). (**B**) Western blot of VEGF in ER-positive Ishikawa cells and ER-negative Ishikawa02 cells after treatment with E2 and E2 plus raloxifene (R) for 24 hrs. β-actin was used as a loading control. DMSO was added as a vehicle control for experiments. (**C**) Western blot of VEGF in HUVEC cells after treatment with E2 for 24 hrs. β-actin was used as a loading control.

To investigate the mechanism of oestrogen-induced angiogenesis in adenomyosis, we used the endometrial epithelial cells lines ER-positive Ishikawa and ER-negative Ishikawa02 as the model with reference to previous studies [5,41–45]. First we investigated whether E2 is able to induce VEGF expression in Ishikawa cells. We determined the most suitable dose of E2 in inducing angiogenesis and showed that 10^−6^ M E2 significantly induced VEGF (Fig. S4). In the ER-harbouring cell line Ishikawa, E2 induced VEGF secretion, and raloxifene, a selective ER modulator, abrogated E2-induced VEGF secretion (Fig. 3A). Consistently, E2 also up-regulated VEGF expression and raloxifene abrogated E2-induced VEGF expression in Ishikawa cells (Fig. 3B). In contrast, the effect of E2 in inducing VEGF expression and secretion was not observed in the ER-negative cell line Ishikawa02 (Fig. 3A and B). Next, we investigated whether E2 also induces VEGF expression in the HUVEC. Interestingly, E2 treatment also up-regulated VEGF expression in HUVEC cells (Fig. 3C). The above data indicate that E2 treatment induces VEGF expression in both ER-positive endometrial epithelial cells and endothelial cells.

### Oestrogen promotes the tubal formation and the migration of endothelial cells, and this effect is abrogated by raloxifene or bevacizumab

**Fig. 4:**
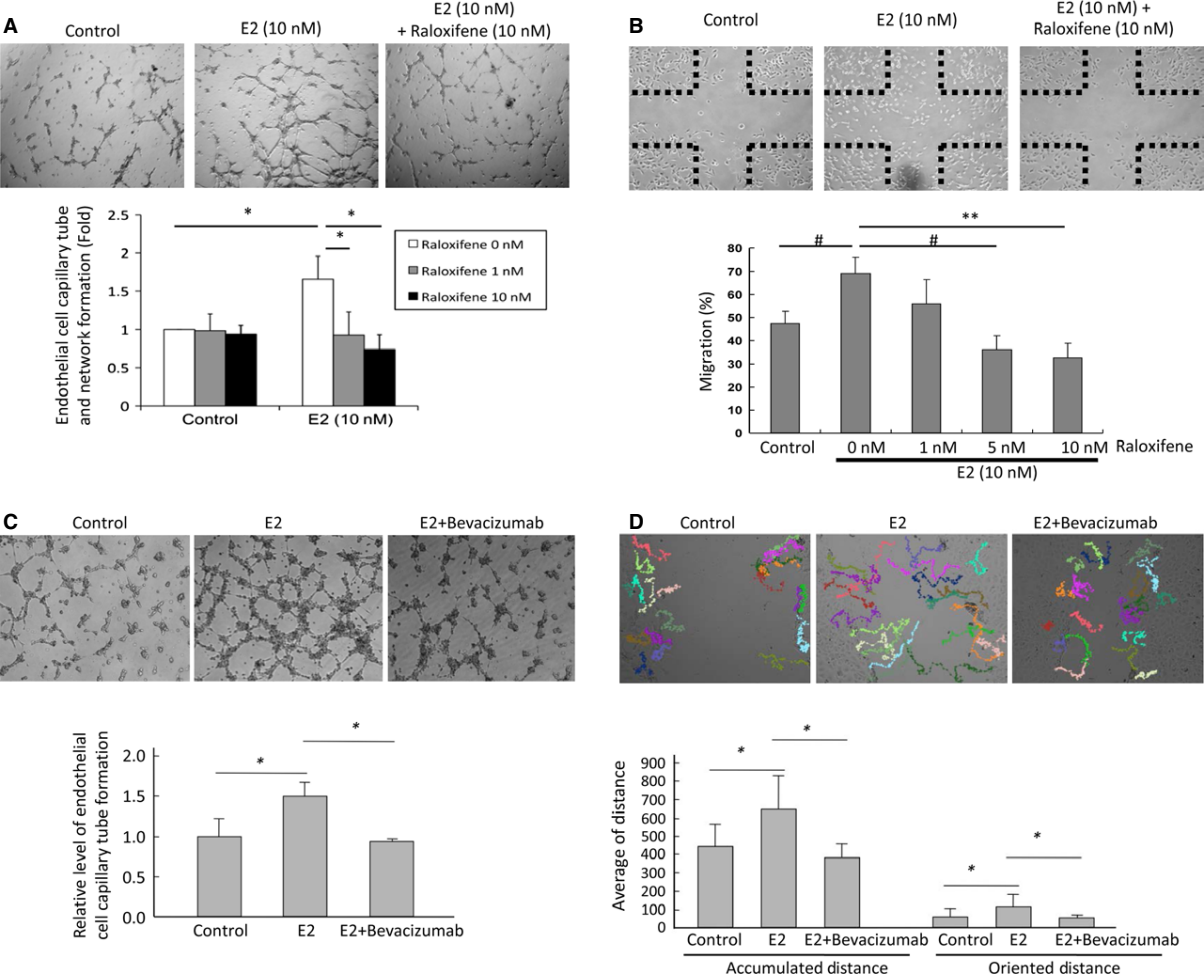
Raloxifene or bevacizumbab inhibits E2-induced endothelial cell tube formation and migration. (**A**) Tube and network formation assay of the HUVEC cells treated with E2 (10 nM) with or without the addition of raloxifene (10 nM). Upper: representative photos. Lower: The levels of endothelial cell tube and network formation were quantified by measuring the total lengths of capillary tubes. The data represent the mean ± SD of three independent experiments. The asterisk (*) indicates statistical significance (*P* < 0.05) by Student's *t*-test. (**B**) *In vitro* wound-healing assay of HUVEC cells treated with E2 with or without the addition of raloxifene. Upper: representative photos. Lower: quantification of the results. The data represent the mean ± SD of three independent experiments. The # indicates statistical significance (*P* < 0.01) by Student's *t*-test. The asterisks (**) indicate statistical significance (*P* < 0.001). (**C**) Tube and network formation assay of HUVEC cells treated with E2 (10 nM), with or without the addition of bevacizumab (0.5 mg/ml). Upper: representative photos. Magnification: 200×. Lower: quantification of the results. The levels of vascular endothelial cell capillary tube and network formation were quantified by measuring the total length of capillary tubes and were normalized to the level of the control. The asterisk (*) indicates statistical significance (*P* < 0.05) by Student's *t*-test. (**D**) *In vitro* wound-healing assay of HUVEC cells treated with E2 (10 nM), with or without the addition of bevacizumab (0.5 mg/ml). Upper: representative trajectories. Cell migration tracks were monitored for 16 hrs by time-lapse photography. Lower: Quantification of the migration distances. The accumulated and oriented migration distances of the HUVECs were quantified by Image-Pro Plus software. The data are expressed as the mean ± SD of three independent experiments, and differences were evaluated by Student's *t*-test. The asterisk (**) indicates statistical significance (*P* < 0.001).

To investigate the impact of oestrogen on promoting angiogenesis, we conducted the capillary tube formation assay and migration assay in HUVEC as HUVEC is a well-established model for studying angiogenesis [46–50]. First, we evaluated the impact of E2 treatment on vascular endothelial cell capillary tube and network formation, and if raloxifene blocks E2-induced tubal formation and migration of HUVEC. The results demonstrated that 10 nM of E2 was able to induce vascular endothelial cell capillary tube and network formation, and this effect was abolished by raloxifene (10 nM; Fig. 4A). Furthermore, to evaluate the influence of E2 on the migratory ability of vascular endothelial cells*,* an *in vitro* wound-healing assay was performed. E2 was able to induce HUVEC migration, and raloxifene inhibited this effect in a dose-dependent manner (Fig. 4B).

We next confirmed whether the neutralization of VEGF can block E2-induced pro-angiogenic activity. The capillary tube and network formation assay was performed in E2-treated HUVEC cells with or without bevacizumab, a VEGF neutralizing antibody. E2-induced capillary formation was abrogated by bevacizumab (Fig. 4C). We further examined the inhibitory effect of bevacizumab on HUVEC migration. Treatment with bevacizumab significantly reduced E2-induced HUVEC migration in both the oriented distance and the accumulated distance (Fig. 4D). The above results suggest that both raloxifene and bevacizumab are able to abrogate the E2-induced pro-angiogenic activity of endothelial cells.

### Slug is responsible for oestrogen-induced VEGF expression in endometrial epithelial cells and subsequent angiogenesis

**Fig. 5:**
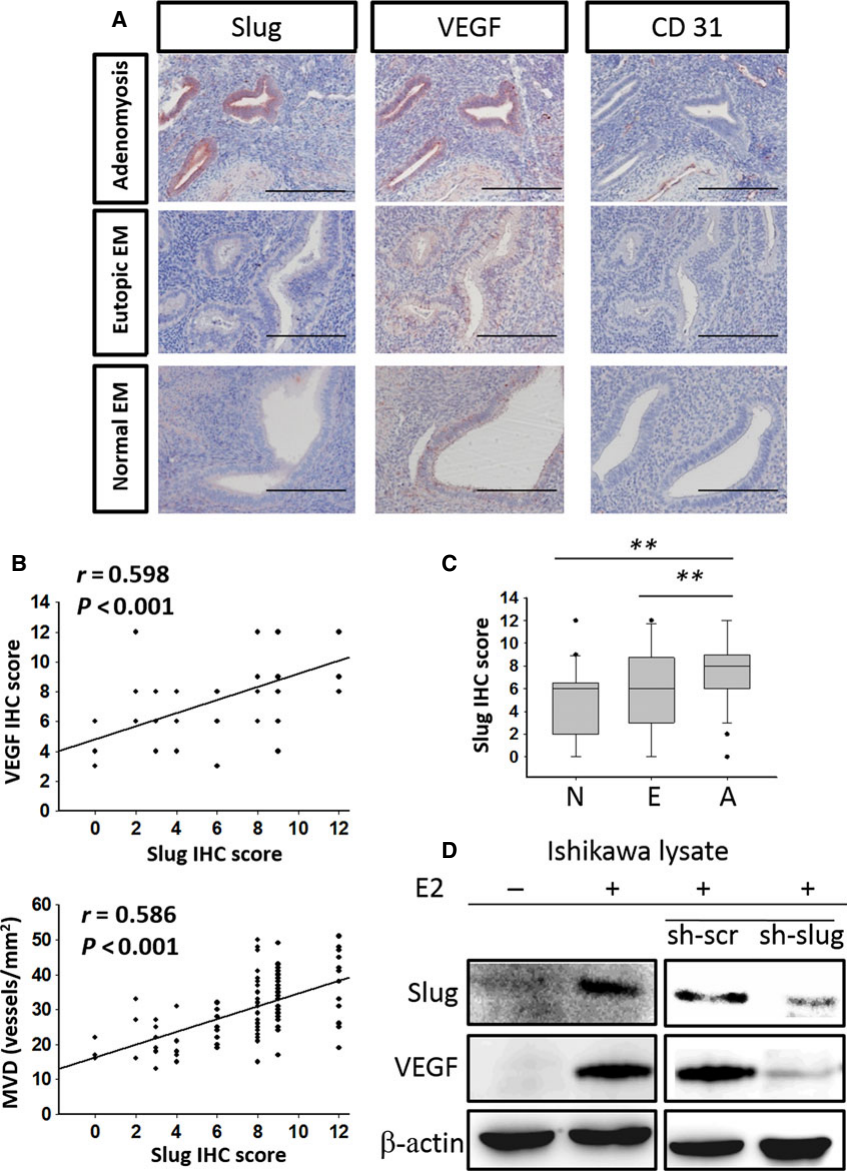
Slug correlates with angiogenesis markers in adenomyosis and is responsible for VEGF expression in endometrial epithelial cells. (**A**) Immunohistochemistry of Slug, VEGF and CD31 in normal endometria, eutopic endometria and adenomyosis. The scale bars represent 800 μm. Original magnification: ×400. (**B**) Correlation between Slug, VEGF and MVD in adenomyotic tissue of patients with adenomyosis (linear regression test). The *P*-value and correlation coefficient R are presented in each panel. (**C**) The boxplots presenting the Slug IHC score in normal endometria (N), eutopic endometria (E) and adenomyosis (A). The asterisks (**) indicate statistical significance (*P* < 0.001). (**D**) Western blot of Slug and VEGF in Ishikawa ER+ cells with/without E2 stimulation expressing a shRNA against Slug or a scrambled control. β-actin was used as a loading control.

Epithelial–mesenchymal transition has been shown to induce angiogenesis in different human cancers, and [51] the EMT regulators (Slug, Twist and Snail) are critical in angiogenesis [51–54]. We previously show that E2 induces EMT in endometrial epithelial cells through Slug [5]. Slug has also been shown to be involved in endothelial cell proliferation and angiogenesis [52–56]. We herein hypothesized that Slug is critical in promoting angiogenesis of adenomyosis. First, we examined the correlation between Slug and the angiogenesis markers VEGF and MVD in normal endometria, eutopic endometria and adenomyosis tissues. A positive correlation among the IHC score of Slug and VEGF (*r* = 0.598, *P* < 0.001) was observed only in adenomyosis tissues but not in normal endometria and eutopic endometria (Fig. 5A and B). A positive correlation among the IHC score of Slug and MVD (*r* = 0.586, *P* < 0.001) was also found only in adenomyosis tissues but not in normal endometria and eutopic endometria (Fig. 5A and B). These results suggest that the Slug-induced EMT of endometrial epithelial cells is associated with an increased angiogenesis in adenomyotic tissue. Compared with normal endometria, eutopic endometria exhibited a higher Slug-positive rate, and the adenomyotic tissues exhibited the highest rate (Fig. 5C; *P* < 0.05). A higher Slug expression was also shown in the secretory phase of eutopic endometrium and adenomyotic tissues compared with the proliferative phase (Fig. S2G–I). These results suggest that the Slug is possible key factor in adenomyotic tissue. The *in vitro* result further confirms the role of Slug in oestrogen-induced angiogenesis: in ER-harbouring Ishikawa cells, knockdown of Slug abrogated E2-induced VEGF expression (Fig. 5D). These results suggest that Slug is the major factor responsible for E2-induced VEGF expression and angiogenesis in adenomyosis.

### Oestrogen induces implantation of adenomyotic lesions and angiogenesis in ovariectomized SCID mice, and raloxifene or bevacizumab abrogates such effects

**Fig. 6:**
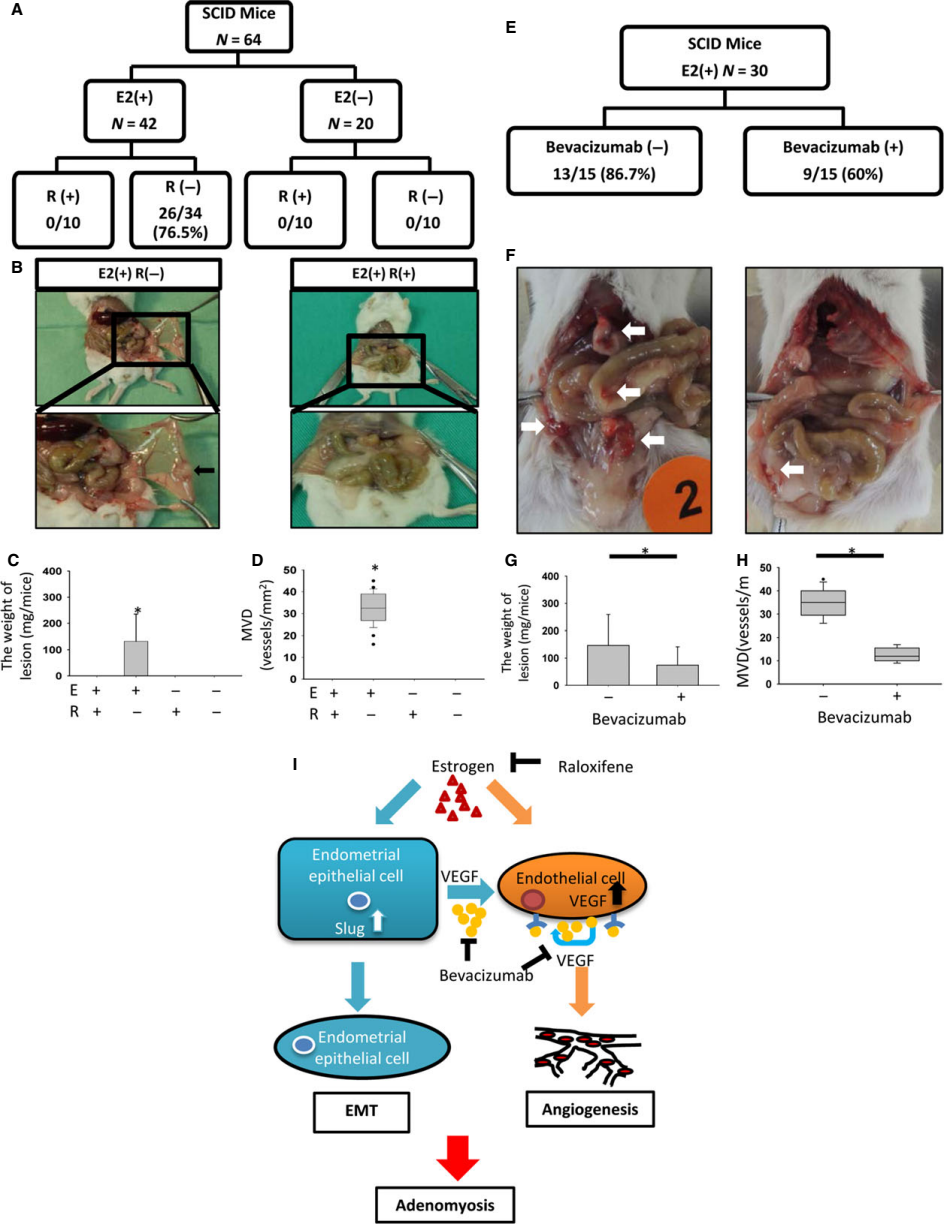
Raloxifene or bevacizumab inhibits E2-induced implantation of adenomyotic lesions, and a hypothetic model E2-induced angiogenesis in adenomyosis through epithelial–mesenchymal transition (EMT). (**A**) The flow chart of the experiments. Sixty-four mice transplanted with adenomyotic lesions in the early proliferative phase were separated into four groups: E2 (+) R (+), E2 (+) R (−), E2 (−) R (+) and E2 (−) R (−). E2, estradiol; R, raloxifene. (**B**) Representative images of the implanted fragments of adenomyotic tissues into the peritoneal cavity of the mice. The arrows indicate the implanted endometrial tissues and neovascular formation. (**C**) A histogram demonstrating the weight of the implanted lesions in each group. *P < 0.05. (**D**) A boxplot demonstrating the microvascular density (MVD) of the implanted lesions in each group. *P < 0.05. (**E**) The flow chart of the experiments. Thirty mice transplanted with adenomyotic lesions in the early proliferative phase were separated into two groups: bevacizumab (+) *versus* bevacizumab (−). All mice were treated with E2 to facilitate the implantation of adenomyotic lesions. (**F**) Representative pictures of the implanted fragments of adenomyotic tissues into the peritoneal cavity of the mice. The arrows indicate the implanted endometrial tissues and neovascular formation. (**G**) A histogram demonstrating the weight of the implanted lesions in each group. *P < 0.05. (**H**) A boxplot demonstrating the MVD of the implanted lesions in each group. *P < 0.05. (**I**) A hypothetical model of E2-induced angiogenesis in adenomyosis through EMT.

Finally, we confirmed the role of E2 and VEGF in the increased adhesiveness and angiogenesis of adenomyotic lesions *in vivo*. First, we performed ovariectomies in 64 SCID mice. After 2 weeks, the adenomyotic tissues were implanted into the peritoneal cavity of female SCID mice. After xenotransplantation, the mice were divided into four groups receiving different treatments: vehicle control, E2, E2+ raloxifene or raloxifene alone. The algorithm used for the animal experiments is illustrated in Figure 6A. In mice with adenomyotic lesion transplants, only those treated with E2 developed viable adenomyotic lesions and neovessels after a 21-day incubation (Fig. 6B and C). IHC analysis revealed that VEGF was increased in the endometrial epithelial cells of viable adenomyotic lesions. Significant vascularity demonstrated by CD31 IHC was observed in the stroma of viable lesions (Fig. S5, Fig. 6D). In contrast, there were no visible adenomyotic lesions or neovessels in the control, raloxifene and E2+ raloxifene groups. These results suggest that oestrogen significantly enhances the adhesiveness and angiogenesis of adenomyotic lesions and that such effects were suppressed by raloxifene. Next, we determined the role of VEGF in E2-induced adenomyosis. A similar approach was performed in 30 ovariectomized SCID mice, and all mice were treated with E2. Fifteen mice were treated with the VEGF blocker bevacizumab, and 15 mice were treated with vehicle control. The algorithm is illustrated in Figure 6E, and the representative photos are in Figure 6F. Bevacizumab significantly reduced but did not abrogate the E2-induced implantation of adenomyotic tissues. Furthermore, bevacizumab also reduced the gross vascularity (Fig. 6F) and weight of the implanted lesion (Fig. 6G). Microscopically, bevacizumab attenuated the MVD of implanted lesions and VEGF expression in endometrial epithelial cells (Fig. S6 and Fig. 6H).

According to the present data, we propose a model to summarize our findings (Fig. 6I). In adenomyosis patients, up-regulation of E2 levels induces angiogenesis by increasing VEGF expression in both endometrial epithelial cells and endothelial cells. In endometrial epithelial cells, up-regulation of Slug by E2 induces EMT and produces VEGF to promote angiogenesis. Using raloxifene to antagonize the E2 effect or bevacizumab to neutralize VEGF reduces angiogenesis in adenomyosis.

## Discussion

The major characteristics of adenomyotic cells include their steroidogenic potential, increased angiogenesis and increased migration and invasion [4,5]. However, it remains unclear how oestrogen promotes angiogenesis, and the correlation between oestrogen-induced angiogenesis and endometrial cells migration in adenomyosis is unknown. In this study, we demonstrated that oestrogen promotes endometrial angiogenesis by inducing VEGF expression in both glandular epithelial cells and endothelial cells, which results in new blood vessel formation. Intriguingly, oestrogen is able to induce migration and VEGF expression in both endometrial epithelial cells and vascular endothelial cells. Our data highlight the crucial role of oestrogen-induced EMT in the development of adenomyosis through the autocrine and paracrine effects of VEGF.

Slug is a zinc finger transcriptional factor and has been recognized as a major EMT inducer through repressing E-cadherin expression [57]. We previously demonstrated that Slug is responsible for oestrogen-induced EMT in endometrial epithelial cells [5]. In this study, we further confirmed the role of Slug in oestrogen-induced VEGF expression of the endometrial epithelial cells, and a positive correlation between the expression of Slug and VEGF and MVD was demonstrated in adenomyotic tissues of patients. Interestingly, in comparison with the early proliferative phase, a significant higher IHC score of Slug and VEGF were observed in the secretive phase of eutopic endometria and adenomyosis tissues. This result confirms the increased angiogenic activity in response to oestrogen levels in normal endometria discovered by previous studies [58]. Furthermore, the pathogenic role of EMT in adenomyosis through promoting both the migration of endometrial cells and angiogenesis is therefore revealed. However, EMT may only be one of the pathways to induce adenomyosis as the evidence for directly supporting its role in adenomyosis development is relatively lacking. Further experiments for confirming the effect of Slug in adenomyosis development, such as reconstitution of Slug in the implanted endometrial cells in ovariectomized SCID mice treated with E2 and raloxifene, will be necessary.

To relieve secondary dysmenorrhoea caused by adenomyosis, hysterectomy is the major strategy. However, fertility and uterus preservation is compromised by such treatment. Removing adenomyotic lesions instead of hysterectomy is another strategy to treat adenomyosis; however, the effect is temporary, and most women quickly develop adenomyosis and must undergo hysterectomy within 1 year. Even if a normal pregnancy is achieved after removing adenomyotic lesions, safety remains a major concern during pregnancy. Residual adenomyosis in the myometrium combined with the possible inhibition of pregnancy-related changes, such as uterine softening, may increase the risk of miscarriage or uterine rupture. Traditional pharmacological therapies for adenomyosis are primarily aimed at the suppression of endogenous oestrogen production by the application of the GnRH agonists and low-dose oral contraceptives. However, the results are unsatisfying [59]. Thus, there is an urgent need to develop novel treatment strategies for adenomyosis. In our study, we provide the rationale for the stepwise blocking strategy of angiogenesis to treatment adenomyosis. First, we demonstrated that raloxifene, a selective ER modulator that agonizes or antagonize ER in a tissue-selective manner, suppresses oestrogen-induced EMT and angiogenesis. Second, neutralizing VEGF by bevacizumab suppressed oestrogen-induced angiogenesis *in vitro* and the implantation of adenomyotic lesions *in vivo*, suggesting that it may be an appropriate drug for the treatment of adenomyosis. Using this novel strategy to block the major pathogenic process of adenomyosis will be a promising strategy to alleviate the symptoms of adenomyosis while preserving fertility.

In our study, we focus on the role of oestrogen-induced adenomyosis through promoting angiogenesis. The limitation of our study is that we did not consider the impact of progesterone on angiogenesis as the peak progesterone secretion is in the mid-secretory phase [60]. Few studies have investigated the effects of progesterone on normal endometrial angiogenesis [61]. Progesterone inhibited proliferation and arrested the cell cycle of human dermal endothelial [62,63]. Furthermore, progesterone inhibited expression of matrix metalloproteinases and angiogenic factors and suppress microvessel density in human ectopic endometrial lesions [64]. Together with our finding, we suggest that oestrogen and progesterone may have opposite effects in promoting angiogenesis in adenomyotic lesions. Another limitation of our study is that it is difficult to completely separate the endometrium from surrounding myometrium even we confirmed that the major component of the implanted lesions is ectopic endometrium rather than myometrium by microscopy before implantation. However, the lesions successfully implanted into the peritoneum of mice and the histology further confirms the implanted endometrium. We therefore suggest that although this model is not able to completely separate the endometrium from myometrium, it is still a suitable model for investigating the invasive behaviour of adenomyotic lesions.

In conclusion, our study demonstrates the pivotal role of oestrogen-induced EMT in the pathogenesis of adenomyosis; EMT not only enhances the migration of endometrial epithelial cells but also orchestrates the angiogenesis process through the autocrine and paracrine effect of VEGF. Furthermore, we also provide a feasible strategy for treating adenomyosis *via* targeting both E2 and VEGF to reverse oestrogen-induced EMT and angiogenesis. The synergistic effect of combination treatment will be very promising but requires further study for validation.

## Supporting information

**Figure S1.** Quantification of the vascularity index (VI) and vascularity flow index (VFI) detected by 3D power Doppler sonography in women with or without adenomyosis during different menstrual phases. **Figure S2.** The boxplots for the IHC scores of VEGF (A–C), MVD (D–F) and Slug (G–I) in normal endometria (A, D, G), eutopic endometria (B, E, H) and adenomyosis (C, F, I) in different menstrual phases. **Figure S3.** Correlation between serum E2 levels and angiogenesis markers in normal endometria, eutopic endometria and adenomyotic lesions. **Figure S4.** Western blot of the VEGF in Ishikawa cells after treatment with the different concentration of E2 for 24 hrs. **Figure S5.** Haematoxylin & Eosin stain (HE) and the immunohistochemistry of VEGF and CD31 of the implanted adenomyotic lesions in the E2+ raloxifene animal experiments (see Fig. 6A–D). **Figure S6.** Haematoxylin & Eosin stain (HE) and the immunohistochemistry of VEGF and CD31 of the implanted adenomyotic lesions in the E2+ bevacizumab animal experiments (see Fig. 6E–H). **Table S1.** List of proteins tested by antibodies and characteristics of the corresponding antibodies used. **Table S2.** Sequence of the oligonucleotides for shRNA construct making. **Table S3.** Distribution of menstrual phases in samples from patients with adenomyosis and normal control. **Table S4.** Serum E2 level in different menstrual phases of adenomyosis patients and normal control cases.
