# Supplementary material for: Oestrogen-induced angiogenesis promotes adenomyosis by activating the Slug-VEGF axis in endometrial epithelial cells
Source: J Cell Mol Med. 2014 Apr 24;18(7):1358–71. doi: 10.1111/jcmm.12300 (PMC4124020; doi:10.1111/jcmm.12300)
Supplement: Supplementary file 1 [file jcmm0018-1358-SD1.doc]

**Supporting Information**

**Estrogen-induced angiogenesis promotes adenomyosis by activating the Slug-VEGF axis in endometrial epithelial cells**

Tze-Sing Huang, Yi-Jen Chen*, Teh-Ying Chou, Chih-Yao Chen, Hsin-Yang Li, Ben-Shian Huang, Hsiao-Wen Tsai, Hsin-Yi Lan, Cheng-Hsuan Chang, Nae-Fang Twu, Ming-Shyen Yen, Peng-Hui Wang, Kuan-Chong Chao, Chun-Chung Lee, and Muh-Hwa Yang

**Table of content**

**Figure S1**

**Figure S2**

**Figure S3**

**Figure S4**

**Figure S5**

**Figure S6**

**Table S1**

**Table S2**

**Table S3**

**Table S4**


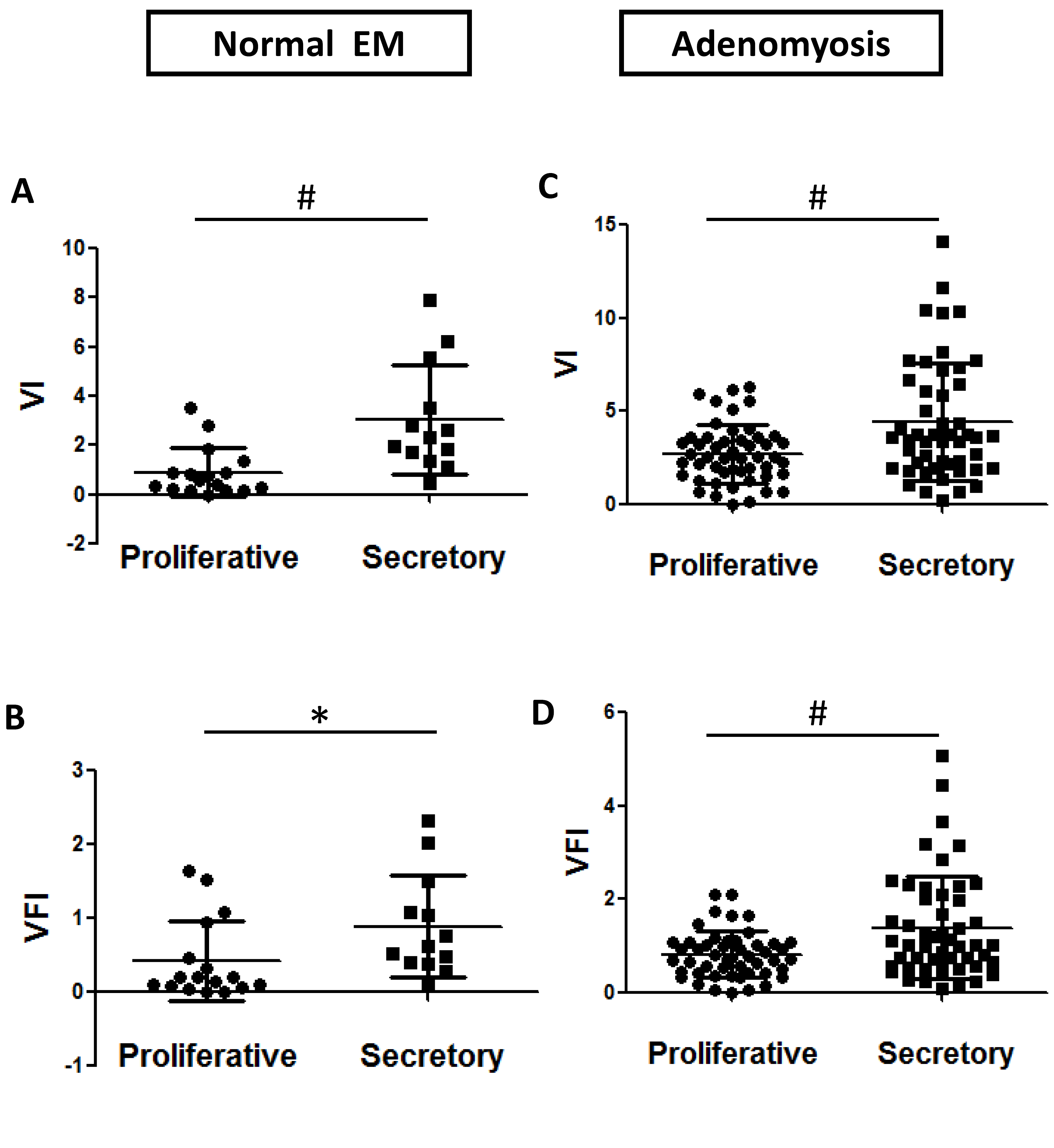


**Figure S1.** Quantification of the vascularity index (VI) and vascularity flow index (VFI) detected by 3D power Doppler sonography in women with or without adenomyosis during different menstrual phases. The # indicates statistical significance (*P* <0.01) by Student’s *t*-test. The asterisk (*) indicates statistical significance (*P* < 0.05) between the different phases.

**Figure S2.** The boxplots for the IHC scores of VEGF (A-C), MVD (D-F), and Slug (G-I) in normal endometria (A,D,G), eutopic endometria (B,E,H), and adenomyosis (C,F,I) in different menstrual phases. P: proliferative phase; S, secretory phase. The asterisk (*) indicates statistical significance (P<.05) by Student’s *t*-test. The # indicates statistical significance (*P* < 0.01) between the different phases. The double asterisk (**) indicates statistical significance (*P* < 0.001).

**Figure S3.** Correlation between serum E2 levels and angiogenesis markers in normal endometria, eutopic endometria, and adenomyotic lesions. A-C, The histograms demonstrating the E2 levels in VEGF IHC-positive vs. VEGF IHC-negative cases in normal endometria (A), eutopic endometria (B), and adenomyosis (C). The bars represent the mean value ± SD. D-F, The linear regression model for demonstrating the correlation between microvascular density (MVD) and E2 levels in normal endometria (D), eutopic endometria (E), and adenomyosis (F). The P value and correlation coefficient R are presented in each panel.

**Figure S4.** Western blot of the VEGF in Ishikawa cells after treatment with the different concentration of E2 for 24 h. β-actin was used as a loading control. The quantification of the western blot results is shown along with the electrophoretic gel bands.

**Figure S5.** Hematoxylin & Eosin stain (HE) and the immunohistochemistry of VEGF and CD31 of the implanted adenomyotic lesions in the E2 + raloxifene animal experiments (see Figure 6A-D). The representative samples are from the E2 (+) R (-) group. The scale bars represent 800 μm. Original magnification: x100 (Left); x400 (Right).

**Figure S6**. Hematoxylin & Eosin stain (HE) and the immunohistochemistry of VEGF and CD31 of the implanted adenomyotic lesions in the E2 + bevacizumab animal experiments (see Figure 6E-H). The representative samples are from the bevacizumab (+) vs. bevacizumab (-) group. The scale bars represent 800 μm. Original magnification: x100 (Left).

**Table S1**. List of proteins tested by antibodies and characteristics of the corresponding antibodies used

| Protein | Assay | Antibody | Origin | Dilution | Incubation period |
| --- | --- | --- | --- | --- | --- |
| VEGF | IHC | sc-152, Santa Cruz | rpab | 1/200 | overnight, 4°C |
| VEGF | WB | sc-152, Santa Cruz | rpab | 1/500 | overnight, 4°C |
| CD31 | IHC | M8023 (clone JC70A), Dako | mmab | 1/100 | overnight, 4°C |
| Slug | IHC | No. 9589, Cell Signaling  Technology, Inc | mmab | 1/100 | overnight, 4°C |
| Slug | WB | No. 9589, Cell Signaling  Technology, Inc | mmab | 1/1000 | overnight, 4°C |
| Akt | WB | No. 9272, Cell Signaling | rpab | 1/1000 | overnight, 4°C |
| p-Akt | WB | No. 9271, Cell Signaling | rpab | 1/1000 | overnight, 4°C |
| Erk | WB | No. 9102, Cell Signaling | rpab | 1/1000 | overnight, 4°C |
| p-Erk | WB | No. 05-797, Millipore | rmab | 1/1000 | overnight, 4°C |
| β-actin | WB | A1978,Sigma, Inc. | mmab | 1/10000 | overnight, 4°C |

Abbreviations: IHC, immunohistochemistry; mmab, mouse monoclonal antibody; rpab, rabbit polyclonal antibody; rmab, rabbit monoclonal antibody; WB, Western blot

**Table S2. Sequence of the oligonucleotides for shRNA construct-making.**

| shRNA | sequence |
| --- | --- |
| sh-Slug | GATCCCCGATGCATATTCGGACCCACTTCAAGAGAGTGGGTCCGAATATGCATCTTTTTA |
| sh-scramble | GATCCCCGTGTCTGTAGGAGTCATCCTTCAAGAGAGGATGACTCCTACAGACACTTTTTA |

**Table S3. Distribution of menstrual phases in samples from patients with**

**adenomyosis and normal control**

| Variables | Normal endometrium | Adenomyosis | P |
| --- | --- | --- | --- |
| Case No. | 30 | 100 |  |
| Age | 46.50±4.49 | 45.15±4.51 | .153 |
| Early and mid-proliferative phase | 17(56.7%) | 50(50%) |  |
| Secretory phase | 13(43.3%) | 50(50%) |  |
| Uterine volume (cm^3^) | 125.41±58.98 | 365.41±256.32 | <.001 |
| Ca125 (U/ml) | 20.72±27.49 | 85.45±146.51 | <.001 |
| Hgb (g/dl) | 12.33 ±1.81 | 11.08±1.56 | <.001 |
| Dysmenorrhagia | 8(26.7%) | 65(65%) | <.001 |
| Menorrhagia | 11(36.7%) | 65(65%) | 0.006 |

Mean ± SD, standard deviation.

**Table S4. Serum E2 level in different menstrual phases of adenomyosis patients and normal control cases**

|  | Menstrual phases | Mean value of serum E2  (pg/ml ± SD) | *p* value  (between menstrual phases) |
| --- | --- | --- | --- |
| Adenomyosis | Early and mid proliferative proliferative (n=50) | 31.57±22.05 | <0.001* |
|  | Secretory (n=50) | 87.43 ±90.22 |  |
| Normal control | Early and mid proliferative proliferative (n=17) | 23.85 ±9.38 | 0.005* |
|  | Secretory (n=13) | 54.76±40.87 |  |

* Statistical significance.
